# Tocilizumab (TCZ) Decreases Angiogenesis in Rheumatoid Arthritis Through Its Regulatory Effect on miR-146a-5p and EMMPRIN/CD147

**DOI:** 10.3389/fimmu.2021.739592

**Published:** 2021-12-15

**Authors:** Devy Zisman, Mirna Safieh, Elina Simanovich, Joy Feld, Amalia Kinarty, Liron Zisman, Tal Gazitt, Amir Haddad, Muna Elias, Itzhak Rosner, Lisa Kaly, Michal A. Rahat

**Affiliations:** ^1^ Ruth and Bruce Rappaport Faculty of Medicine, Technion-Israel Institute of Technology, Haifa, Israel; ^2^ Department of Rheumatology, Carmel Medical Center, Haifa, Israel; ^3^ Immunotherapy Laboratory, Carmel Medical Center, Haifa, Israel; ^4^ Rheumatology Unit, Bnei Zion Medical Center, Haifa, Israel

**Keywords:** rheumatoid arthritis (RA), tocilizumab (TCZ), angiogenesis, EMMPRIN/CD147, miR-146a-5p, thrombospondin-1 (Tsp-1)

## Abstract

**Background:**

Angiogenesis is a major contributor to the development of inflammation during Rheumatoid arthritis (RA), as the vascularization of the pannus provides nutrients and oxygen for the infiltrating immune cells and proliferating synoviocytes. Tocilizumab (TCZ) is an anti-IL-6 receptor antibody that is used in the treatment of RA patients, and has been shown to exert anti-inflammatory effects. However, its effects on angiogenesis are not fully elucidated, and the molecular mechanisms regulating this effect are unknown.

**Methods:**

We evaluated the concentrations of several pro- and anti-angiogenic factors and the expression levels of several microRNA molecules that are associated with RA and angiogenesis in serum samples obtained from 40 RA patients, before and 4 months after the initiation of TCZ treatment. Additionally, we used an *in vitro* co-culture system of fibroblasts (the HT1080 cell line) and monocytes (the U937 cell line) to explore the mechanisms of TCZ action.

**Results:**

Serum samples from RA patients treated with TCZ exhibited reduced circulating levels of EMMPRIN/CD147, enhanced expression of circulating miR-146a-5p and miR-150-5p, and reduced the angiogenic potential as was manifested by the lower number of tube-like structures that were formed by EaHy926 endothelial cell line. *In vitro*, the accumulation in the supernatants of the pro-angiogenic factors EMMPRIN, VEGF and MMP-9 was increased by co-culturing the HT1080 fibroblasts and the U937 monocytes, while the accumulation of the anti-angiogenic factor thrombospondin-1 (Tsp-1) and the expression levels of miR-146a-5p were reduced. Transfection of HT1080 cells with the miR-146a-5p mimic, decreased the accumulation of EMMPRIN, VEGF and MMP-9. When we neutralized EMMPRIN with a blocking antibody, the supernatants derived from these co-cultures displayed reduced migration, proliferation and tube formation in the functional assays.

**Conclusions:**

Our findings implicate miR-146a-5p in the regulation of EMMPRIN and propose that TCZ affects angiogenesis through its effects on EMMPRIN and miR-146a-5p.

## Introduction

### Tocilizumab (TCZ) in RA Treatment

Rheumatoid arthritis (RA) is a chronic autoimmune disease that causes joint inflammation, damage and bone erosion, as well as many systemic manifestations. The pathophysiology of RA is based on a complex network of pro-inflammatory cytokines, especially interleukin-6 (IL-6) and tumor necrosis factor-α (TNFα) (1, 2). The binding of IL-6 to its membranal receptor or to its soluble receptor and the subsequent binding of the complex to the gp130 receptor chains evokes signals, primarily through JAK/STAT3 activation, but also through the activation of the MAPKs and PI3K/Akt pathways (3) that give rise to the pleotropic effects of this cytokine. These pathways, together with TNFα and IL-1β, synergistically activate pro-inflammatory and pro-angiogenic molecules (4, 5). This accounts for the pro-inflammatory effects of IL-6 including neutrophil and monocyte recruitment, endothelial cell activation, B cell stimulation leading to autoantibody production, and induction of acute phase reactants such as C-reactive protein (CRP) and serum amyloid A (SAA) (3). Furthermore, IL-6 together with TNFα drive the proliferation of fibroblast-like synoviocytes (FLS) and promote their local secretion of a myriad of cytokines (6), as well as their stimulation of angiogenesis through phosphorylation of STAT3 and induction of vascular endothelial growth factor (VEGF) production (7, 8).

The humanized anti-IL-6 receptor monoclonal antibody tocilizumab (TCZ) blocks both the *cis* and *trans* signaling pathways of IL-6. Although it is known to lead to the accumulation of IL-6 in the serum of treated patients (2), TCZ reduces the serum levels of pro-inflammatory cytokines [e.g., RANKL, MIF, chemerin, IL-21 (9, 10)], reduces CRP and SAA levels, and ameliorates the systemic manifestation of RA, such as pain, fatigue, and anemia (1, 2, 10). While the effects of TCZ on angiogenesis have yet to be investigated in depth, TCZ has been shown to reduce VEGF serum levels in complete Freund’s adjuvant (CFA)-induced arthritic rats (11), and to reduce the mean vessel density (MVD) in the synovium of RA patients as evaluated by immunohistochemical staining for the endothelial marker CD31 (12). However, the detailed mechanisms of action, which enable TCZ to inhibit angiogenesis, are not yet understood.

To allow immune cells to infiltrate, and to supply the growing metabolic needs of these cells and of the proliferating FLS for oxygen and nutrients, new blood vessels must be formed in the expanding pannus. Therefore, angiogenesis is considered critical to pannus formation. The increasing number of cells results in local hypoxia, which stimulates the production of pro-angiogenic factors (e.g., VEGF). Thus, an excess of pro-angiogenic relative to anti-angiogenic mediators is generated, and this imbalance switches on angiogenesis and increases blood vessel density to help sustain pannus progression (7).

Here we investigate the role of the several pro-angiogenic factors: VEGF, which promotes endothelial cell migration, proliferation, and tube formation (13), and is the most potent pro-angiogenic factor known; matrix metalloproteinases (MMPs) which degrade the basement membrane and allow endothelial cell migration, particularly MMP-9; the neutrophil gelatinase associated lipocalin (NGAL), which can bind to and protect MMP-9 from degradation (14); and EMMPRIN/CD147 which can induce both VEGF and MMPs secretion and therefore is considered a pro-angiogenic factor (15). On the other hand, we also study thrombospondin-1 (Tsp-1), which is an example of a potent endogenous inhibitor of angiogenesis (16, 17).

MicroRNAs (miRNAs) are small (20-23 nucleotides long) non-coding RNA molecules, which function in post-transcriptional regulation of gene expression *via* by base-pairing with complementary mRNA sequences leading to their silencing by cleavage or inhibited translation. Thus, miRNAs have been implicated in the regulation of many cellular processes, including angiogenesis, particularly in the context of malignancy (18–22). Several miRNAs have previously been implicated in the pathogenesis of RA, some of which are also known to be involved in the regulation of angiogenesis (18, 20, 23–25). Of these, we selected 9 miRNAs to examine the effect of TCZ on their circulating levels. For instance, we have previously linked the regulation of EMMPRIN expression to miR-146a-5p in cancer cells (26, 27), and miR-146a-5p has been linked to angiogenesis in the context of RA (28). However, little is known about the involvement of miRNAs in angiogenesis in the context of RA.

The goal of the research presented here was to evaluate the effects of TCZ on levels of pro- and anti-angiogenic factors found in sera of RA patients, as well as on the accumulation of several circulating miRNAs known to be associated with angiogenesis. Our findings, including the regulatory role of miR-146a-5p on angiogenesis, were then corroborated in an *in vitro* co-culture system of human monocyte and fibroblast cell lines.

## Methods

### Patients

The study cohort included 40 patients diagnosed with RA, who fulfilled the 2010 EULAR and ACR classification criteria for RA (29) with active disease according to the disease activity score in 28 joints (DAS28-CRP) score (DAS≥3.2) (30) and initiating treatment with tocilizumab infusion (8mg/kg every 4 weeks). The patients were recruited consecutively from the Rheumatology Clinic in Carmel Medical Center and Bnei Zion Medical Center, Haifa, Israel, after failure of conventional disease modifying anti-rheumatic drugs (cDMARDs). All other medical decisions were at the physicians’ discretion. All patients were examined and blood samples were obtained before TCZ infusion at enrollment (referred to as “Before”) and 4 months after the beginning of treatment (referred to as “After 4 m”). Blood was immediately centrifuged, and serum samples were stored at -80°C until further analysis. Response to treatment was assessed according to the EULAR response criteria (30). We further stratified our patients to “responders”- those patients who experienced an improvement in their DAS-28-CRP score ≧ 1.2- and their total DAS28-CRP score was <5.1, and to “non-responders” - patients who demonstrated no change or a change < 1.2 in DAS-28-CRP score (30).

Patients diagnosed with additional inflammatory disease or active neoplastic diseases were excluded from the study. The study was approved by Carmel Hospital Institutional Review Board (Helsinki committee CMC-0018-11) and all patients signed an informed consent form.

### Sandwich Enzyme-Linked Immunosorbent Assay (ELISA)

Concentrations of EMMPRIN, VEGF, MMP-9, IL-6, NGAL, and Tsp-1 were measured using commercial DuoSet ELISA kits (R&D systems, Minneapolis, MN) according to the manufacturer’s instructions. Duplicate serum and supernatant samples were diluted according to preliminary calibration experiments. To deterimine cytokine concentrations in serum samples, samples tested for EMMPRIN, MMP-9 and NGAL were diluted 1:100; samples tested for VEGF and IL-6 were diluted 1:4; and samples tested for Tsp-1 were diluted 1:1000. To determine the cytokine concentrations in supernatants derived from cultured cells, samples were diluted 1:100, except for Tsp-1 (1:1000). The human high sensitivity CRP (hsCRP) was measured by ELISA kit (AssayPro, St. Charles, MO), and samples were diluted 1:4,000.

### Quantitative Real-Time PCR (qPCR) Analyses

Total RNA was extracted from 200 μl of serum derived from the RA patients at the different time points or from 4x10^5^ HT1080 cells, using the total RNA purification kit (Norgen Biotek, Ontario, Canada) according to the manufacturer’s instructions. To assess the expression of specific miRNAs, 350 ng of total RNA were reverse transcribed at 37°C for 1 hour using the High Capacity cDNA Reverse Transcription Kit (Thermo Fisher Scientific/Applied Biosystems, CA) and a mixture of the 5xRT primers for each of the miRNA examined (Thermo Fisher Scientific/Applied Biosystems). The miRNAs measured (miR-16-5p, miR-21-5p, miR-132-3p, miR-146a-5p, miR-150-5p, miR-155-5p, miR-203a-3p, miR-221-3p, and miR-323a-3p) and the RNU6B (U6) small RNA endogenous control were amplified in triplicates using the TaqMan microRNA assay kit (Thermo Fisher Scientific/Applied Biosystems) according to the manufacturer’s instructions. The reaction was carried out for 40 cycles, each of 15 sec at 95°C and 60 sec at 60°C, using the StepOne real-time PCR (Thermo Fisher Scientific/Applied Biosystems). The comparative method (2^-ΔΔCT^) was used for relative quantification, and serum samples from the first visit (before the initiation of TCZ treatment) served as a calibrator in each experiment.

To date, there is still a debate as to which miRNA or small RNA molecule is best to use for normalization strategy for circulating miRNAs (31). Since miR-16 and U6 are two of the most frequently used reference genes, we compared their stability in the serum samples of patients before and 4 months after initiation of TCZ treatment, and chose U6 which demonstrated better stability for the normalization (median U6 C_T_ values of 30.4, IQR 31.4, 28.9; miR-16 median CT value 25.95. IQR 27.91, 23.95) (**Figure S1**).

### Cultured Cells

To study the interactions between fibroblasts and monocytes, we co-cultured the human fibrosarcoma cell line HT1080 (ATCC CCL-12012) and the monocyte-like U937 cells (ATCC 1593). HT1080 cells were cultured in Dulbecco’s modified Eagle’s medium (DMEM, Biological Industries, IL), 10% fetal calf serum (FCS), 1% amphotericin B, 1% L-glutamine, 1% non-essential amino acids (NEAA), and 1% antibiotics, with the addition of 25% conditioned medium supplement derived from the human promyelocytic leukemic cell line HL60 (ATCC CRL-240) which secretes fibroblast growth factor-2 (FGF-2). U937 cells were cultured in RPMI-1640 medium, 10% FCS, amphotericin B (27 μM) and 1% antibiotics (Pen-strep-neomycin). The human endothelial cell line EaHy926 (ATCC CRL-2922) was cultured in DMEM with 10% FCS, 1% glutamine, 2% HAT (mixture of hypoxanthine, aminopterin, and thymidine), and 1% antibiotics. All tissue culture reagents were purchased from Biological Industries, Beit Ha’emek, Israel.

All cell lines were split twice a week at a ratio of 1:4. To avoid the masking of signals, after cells were seeded in plates and allowed to adhere, medium was replaced with serum-starved medium with 0.1% BSA for the duration of the experiment. All cell lines were regularly tested for morphological changes and presence of mycoplasma.

HT1080 (4x10^5^ cells) or U937 (4x10^5^ cells) were cultured separately or in co-culture, with or without the strong MMP-9 inducer TNFα (1ng/mL) or alternatively with recombinant IL-6 (20 ng/ml, PeproTech Asia, Rehovot, Israel), and after 48 hours, supernatants were collected for further analysis. In some experiments, increasing amounts of the recombinant EMMPRIN protein (R&D systems, Minneapolis, MN) was added as indicated, or alternatively, the anti-EMMPRIN blocking antibody (2 ng/ml, Biolegend, San Diego, CA) was added to some of the wells. In other experiments the NF-κB inhibitor Bay 11-7082 (Merck/Sigma-Aldrich, Darmstadt, Germany) or the JAK/STAT inhibitor tofacitinib (Merck/Calbiochem, Darmstadt, Germany) were added to the cultured cells. In experiments where RNA was extracted, HT1080 and U937 cells were separated by an insert (0.4 μm pore size) allowing exchange of soluble nutrients and proteins but precluding cell-cell contact.

### Isolation of Monocyte-Enriched PBMC

Blood (18 ml) was taken from healthy volunteers in the presence of 0.2% EDTA. Peripheral blood mononuclear cells (PBMC) were separated on Histopaque gradient (Sigma-Aldrich, St. Louis, MO) and then washed twice with phosphate-buffered saline (PBS). PBMC were counted and plated in 60-mm dishes at a concentration of 80x10^6^ cells/plate in 3 ml of DMEM medium supplemented with 20% FCS for 3 hours. To enrich for monocytes, non-adherent cells were washed twice with PBS, and monocyte-enriched PBMCs were detached using a scraper and counted again. 8x10^4^ PBMCs were co-cultured with 8x10^4^ HT1080 cells for 48 hours in serum starvation medium with 0.1% BSA, separated by an insert (0.4 μm pore size), with or without the addition of TNFα (1 ng/ml) or IL-6 (20 ng/ml), and in some of the experiments with or without TCZ (500 μg/ml) as described before. This part of the study was approved by Carmel Hospital Institutional Review Board (Helsinki committee CMC-0018-11), and all healthy volunteers signed an informed consent form.

### Wound Assay (*In Vitro*)

EaHy926 cells were seeded (10^5^ cells/well) in 96-well plates and cultured to confluency. The monolayer was then scratched using a toothpick, and the non-adherent cells were removed by washing with PBS. At this point, supernatants from the HT1080 and U937 co-cultures (diluted 1:2) with or without the addition of the anti-EMMPRIN antibody (2 ng/ml), were added to the endothelial layer. Images of the scratch site were acquired immediately after scratching the cell monolayer (T0) and 24 h later (T24) (Moticam 2MP, magnification x4), and the wound area was measured at both times using the ImagePro plus 4.5 software (Media Cybernetics, Inc., Rockville, MD). The subtraction of the area at T0 from the area measured at T24 reflected the area to which the endotheial cells migrated in wound closure.

### Tube Formation Assay (*In Vitro*)

EaHy926 cells (8x10^4^ cells/well) were plated in triplicates in DMEM with 2% FCS on 96-well plates which were previously coated at 4°C with Coultrex^®^ reduced growth factor basement membrane extract (Travigen, Gaithersburg, MD) and polymerized at 37°C for 2 hours. Serum samples (diluted 1:4) or supernatnats from the HT1080 and U937 co-cultures (diluted 1:2) were added. Images were obtained after 6 hours of incubation (Moticam 2MP, magnification x4), and the number of closed lumens were counted in two separate fields.

### Transfection of HT1080 Cells With miR-146-5p Mimic or Anti-miR-146a-5p

HT1080 cells (10^4^ cells) were seeded in a 96-well plate in 100μl of full medium and incubated overnight. The Lipofectamine RNAi MAX (Ambion, Austin, TX) was diluted 1:25 in Opti-MEM medium and combined with an Opti-MEM medium containing 30 nM of miRNA-146a-5p mimic, anti-miR-146a-5p, or their respective negative controls (all from Thermo Fisher Scientific/Ambion) to create miRNA-lipid complex that was added to each well and incubated overnight. Cells were then washed with PBS, and were incubated with serum-starved medium with 0.1% BSA, with or without addition of U937 cells for additional 48 hours, before collecting the supernatants for further analysis.

### Statistics

All values are presented as means ± standard error of measurement (SEM). The nonparametric one-way analysis of variance (ANOVA) test was used to compare multiple groups, followed by the Bonferroni’s multiple post-hoc comparison test. In the patient’s data, two groups were compared with the two-tailed Mann-Whitney U test, or if paired, with the Wilcoxon matched-paired signed rank test. In the *in vitro* experiments, two groups were compared using he unpaired student t test. Detais of each analysis are provided in the figure legends. P values exceeding 0.05 were not considered significant.

## Results

### Study Population

The age of the study population was 57.5 ± 11.1 years, with disease duration of 7.7 ± 5.6 years, 33 (82.5%) were female and 53.9% were positive for rheumatoid factor. The demographic and clinical data of the patients participating in the study are summarized in **Table 1**. All patients continued treatment with TCZ during the study period of 4 months, 25/40 (62.5%) patients were classified as “responders” according to EULAR criteria. Notably, after 4 months of treatment, the mean DAS-28 CRP dropped from 5.47 (IQR 6.1, 4.75) to 3.52 (IQR 4.81, 2.8), tender joint count decreased from 12.5 ± 1.02 to 5.9 ± 0.82, and the swollen joint count decreased from 9.25 ± 0.85 to 4.07 ± 0.66.

**Table 1:** Demographic, clinical characteristics, underlying diseases, and treatment of the study groups.

	Non-responding Patiens	Responding patients	Total RA patients	P values: Responding vs. non-responding
No. participants	15	25	40	
Sex: Female (%)	14 (93.3%)	19 (76%)	33 (82.5%)	ns
Age (years) ± SD	56.9 ± 3.3	57.9 ± 2.1	57.53 ± 11.1	ns
Disease Duration	8 ± 1	7.52 ± 1.3	7.7 ± 5.6	ns
Tobacco use (%)	3 (20%)	7 (28%)	10 (25%)	ns
RF positive (%)	8 (53.3%)	13 (52%)	21 (53.9%)	ns
Anti-CCP positive (%)	3 (20%)	9 (36%)	12 (30%)	ns
**Comorbidities:**				
Hypertension	3 (20%)	9 (36%)	12 (30%)	ns
Hyperlipidemia	8 (53.3%)	11 (44%)	19 (47.5%)	ns
Diabetes mellitus (DM)	5 (33.3%)	3 (12%)	8 (20%)	ns
Chronic obstructive pulmonary disease (COPD)	0 (0%)	1 (4%)	1 (2.5%)	ns
Ischemic heart disease (IHD)	0 (0%)	1 (4%)	1 (2.5%)	ns
Prior malignancy	1 (6.6%)	0 (0%)	1 (2.5%)	ns
**Medications (at baseline):**				
Methotrexate (MTX)	12 (80%)	10 (40%)	27 (67.5%)	0.0217
Sulfasalazine (SSZ)	1 (6.6%)	4 (16%)	5 (12.5%)	ns
Hydroxycholoroquine (HCQ)	1 (6.6%)	3 (12%)	4 (10%)	ns
Leflunomide (LEF)	0 (0%)	3 (12%)	3 (7.5%)	ns
Corticosteroid dose in milligrams (mean + SD)	3 ± 5.9	7.2 ± 10.5	5.62 ± 9.2	ns

Anti-CCP, anti-cyclic citrullinated peptide; ns, not significant; RF, rheumatoid factor; SD, standard deviation.

As expected, serum IL-6 levels were increased after 4 months of treatment (**Figure S2A**) with higher levels in responders than in non-responders (**Figure S2C**). The hsCRP levels were higher in the RA patients before treatment, and were significantly reduced after 4 months of treatment. However, no difference in hsCRP levels was found between responders and non-responders (**Figures S2B, D** **)**.

### TCZ Affects the Concentrations of Pro-Angiogenic Factors

**Figure 1:**
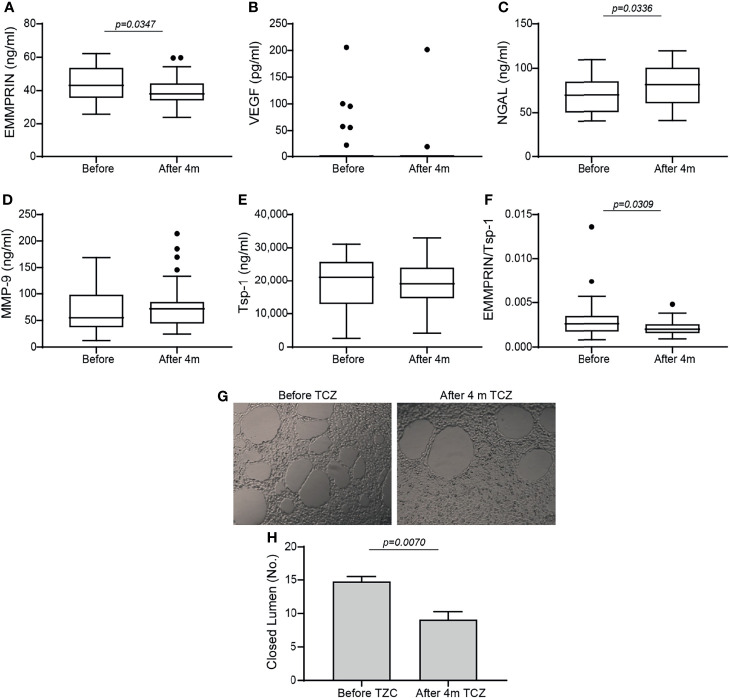
Angiogenic factors in serum samples of RA patients before and 4 months after initiation of TCZ treatment. Box plot representing the concentrations of angiogenic mediators in the serum of RA patients before and 4 months after initiation of TCZ treatment, as determined in duplicates for each sample. **(A)** EMMPRIN, **(B)** VEGF, **(C)** NGAL, **(D)** MMP-9, **(E)** thrombospondin-1 (Tsp-1), and **(F)** the ratio between EMMPRIN and Tsp-1 as a suggested measure of enhanced angiogenesis. The non-parametric Mann-Whitney test was used to compare between the concentrations of each of the cytokines before and 4 months after TCZ treatment (n=40). **(G)** Tube formation assay with **(H)** closed lumen quantitation, to assess the direct angiogenic potential of serum samples on EaHy926 endothelial cell. The tube formation assay was carried out in triplicates with serum samples from selected patients, and the unpaired student *t* test was used to determine the effects of TCZ in the tube formation assay (n=4).

Comparing the levels of pro-angiogenic factors, we noted a drop in EMMPRIN after 4 months of TCZ (**Figure 1A**), an increase in NGAL (**Figure 1C**), and no significant change in MMP-9 or VEGF levels (**Figures 1B, D** **)**. Likewise, no change occurred in the serum levels of MMP-3 and MMP-7 (data not shown). The serum levels of the anti-angiogenetic factors Tsp-1 (**Figure 1E**) and endostatin (data not shown) were also unchanged. Because the angiogenic switch is turned on when the concentrations of pro-angiogenic factors exceed those of anti-angiogenic factors, we calculated the ratio between EMMPRIN (as a pro-angiogenic factor) and Tsp-1 (as an anti-angiogenic factor) for each patient before and 4 months after initiating TCZ, and found a significant decrease following 4 months of treatment (**Figure 1F**).

To show that TCZ affected the balance between serum levels of pro- and anti-angiogenic factors, we assessed their angiogenic potential directly on endothelial cells using the tube formation assay. Serum samples before and 4 months after initiation of TCZ treatment were incubated with the endothelial cell line EaHy926, and the number of closed lumens generated, reflecting the angiogenic potential, was quantified. We show that in accordance with the EMMPRIN levels and the EMMPRIN/Tsp-1 ratio, the endothelial cells generated a reduced number of closed lumens after 4 months of TCZ treatment with thicker layers of cells between the lumens, demonstrating reduced angiogenesis (**Figures 1G, H** **)**.

### Patients Responding to TCZ Treatment Demonstrate Reduced EMMPRIN/Tsp-1 Ratio

**Figure 2:**
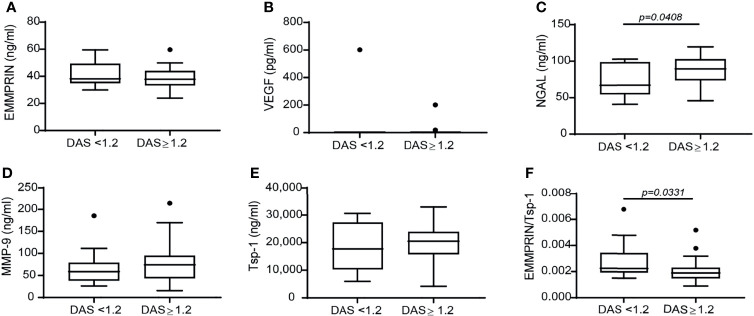
The change in the disease activity score DAS28 is not associated with a difference in the concentration of angiogenic factors, except for the ratio EMMPRIN/Tsp-1. Patients were stratified into responders and non-responders according to the change in their DAS28 score to assess the effects of TCZ on the concentrations of the angiogenic factors. **(A)** EMMPRIN, **(B)** VEGF, **(C)** NGAL, **(D)** MMP-9, **(E)** Tsp-1, and **(F)** the ratio between EMMPRIN and Tsp-1 as a suggested measure of enhanced angiogenesis. The non-parametric Mann-Whitney test was used to compare between the concentrations of each of the cytokines before and 4 months after TCZ treatment (n=40).

To investigate the correlation between the effects of TCZ on angiogenic factors and the clinical response which was observed in treated patients, we stratified the patients into responders and non-responders, resulting in 15 RA patients who did not respond to TCZ treatment and 25 RA patients who responded well to TCZ treatment according to EULAR criteria (30). Only NGAL levels were increased in the responders relative to the non-responders (**Figure 2C**). Levels of VEGF, MMP-9 and Tsp-1, and surprisingly even EMMPRIN levels, were not different between responders and non-responders (**Figures 2A, B, D, E** **)**. However, although each one of these factors separately did not reveal a difference between responders and non-responders, the ratio between EMMPRIN and Tsp-1 was reduced in the responding patients (**Figure 2F**), indicating the usefulness of this ratio in evaluating the angiogenic potential of treated patients.

### TCZ Affects the Serum Expression of miR-146a-5p and miR-150-5p

**Figure 3:**
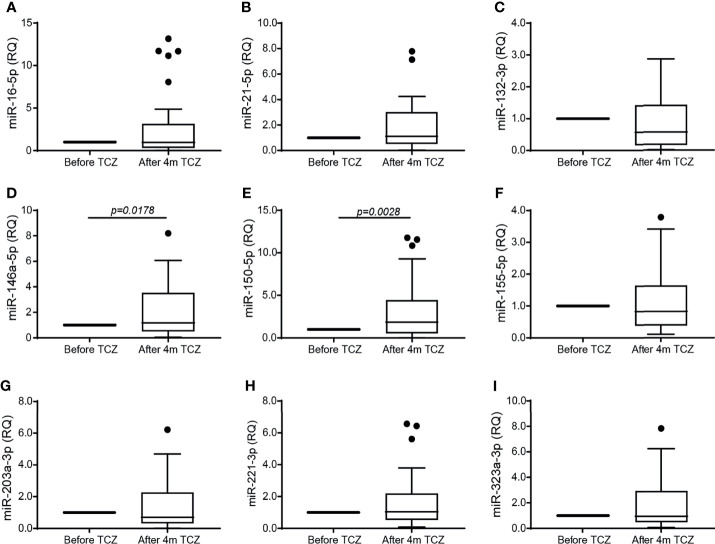
TCZ treatment increases the serum expression of miR-146a-5p and miR-150. Total RNA was extracted from serum samples before and 4 months after initiation of TCZ treatment, and the expression levels of 9 different microRNA known to be involved in angiogenesis and in RA were determined using comparative CT method for relative quantification, where each patient served as its own control. **(A)** miR-16-5p, **(B)** miR-21-5p, **(C)** miR-132-3p, **(D)** miR-146a-5p, **(E)** miR-150-5p, **(F)** miR-155-5p, **(G)** miR-203a-3p, **(H)** miR-223-3p, **(I)** miR-323a-3p. The Wilcoxon matched-paired signed rank test was used to compare between the concentrations of each of the cytokines before and 4 months after TCZ treatment (n=37).

We next asked whether miRNAs are involved in the regulation of angiogenesis in RA and whether TCZ affects their expression. We selected 9 miRNAs whose expression has been linked to angiogenesis in previous studies and which were also shown to have dysregulated expression in RA (25), and followed their expression in RA patients before and after initiation of TCZ treatment. We chose to examine the level of circulating miRNAs, as those are known to be stable and protected from RNase activity within exosomes or when complexed with serum proteins (20). We show that no change occurred in the levels of the miRNAs tested except for the levels of miR-146a-5p and miR-150-5p which were significantly increased after 4 months of TCZ treatment relative to treatment initiation (**Figure 3**). However, no difference was found in the levels of all the serum miRNAs, including miR-146a-5p and miR-150-5p, between patients considered responders and non-responders to TCZ (data not shown).

### EMMPRIN, MMP-9 and VEGF Are Increased, Whereas Tsp-1 Is Reduced in Co-Cultured HT1080 and U937 Cells

**Figure 4:**
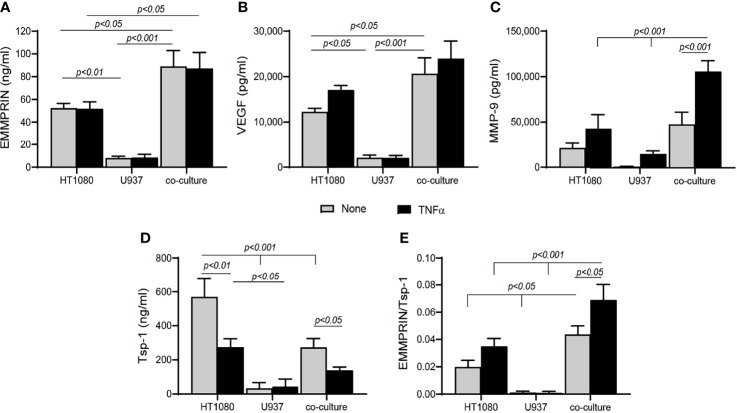
Co-culture enhances angiogenesis. HT1080 cells (4x10^5^ cells/well in 800 μL) were cultured alone or in co-culture with U937 monocytes at a ratio of 1:1, in the absence or presence of TNFα (1ng/mL). Supernatants were collected after 48h of incubation and the concentrations of **(A)** EMMPRIN, **(B)** VEGF, **(C)** MMP-9, and **(D)** Tsp-1 were determined by ELISA. **(E)** The EMMPRIN/Tsp-1 ratio was calculated for each repetition. The two-way analysis of variance (ANOVA) test was used to compare multiple groups, followed by the *post-hoc* Bonferroni’s multiple comparison test (n=6-7 in all groups).

To explore the mechanisms responsible for the changes observed in the patient serum samples after 4 months of TCZ treatment, we turned to an *in vitro* co-culture system of HT1080 fibroblasts and U937 monocytes, similar to our previous study with a different monocytic cell line (32). Levels of secreted EMMPRIN, VEGF and MMP-9 in the U937 single cultures were minimal (**Figures 4A–C**). In co-cultures, EMMPRIN and VEGF levels were synergistically elevated after 48 hours of incubation without TNFα relative to the levels in the single culture of HT1080 (both by about 1.7 fold, p<0.05), whereas MMP-9 levels showed no significant change. The addition of TNFα increased MMP-9 levels in the co-culture (by 2.2 fold, p<0.001), but not those of EMMPRIN or VEGF (**Figures 4A–C**), consistent with the known inducing activity of TNFα on MMP-9 (33). Levels of the anti-angiogenic factor Tsp-1 were reduced in the co-culture relative to the single culture of HT1080 (by 2 fold, p<0.001), and the presence of TNFα further reduced them (by 1.9 fold, p<0.05, **Figure 4D**). The ratio between EMMPRIN and Tsp-1 was increased by the co-culture relative to the single culture of HT1080 cells (by 2 fold, <0.05), and the addition of TNFα further increased it (by 2.3 fold, p<0.05, **Figure 4E**).

### EMMPRIN Expression Promotes VGEF and MMP-9 Expression *In Vitro*, and Neutralization of EMMPRIN Activity Reduces Angiogenesis

**Figure 5:**
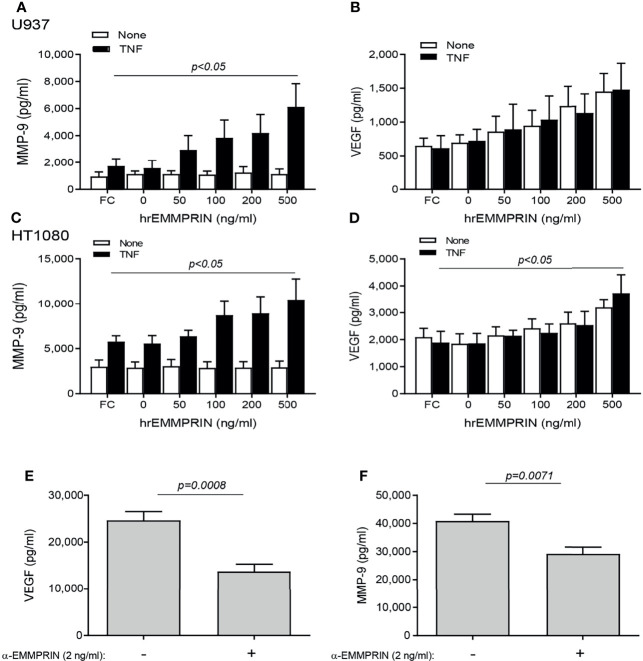
EMMPRIN regulates the increase in VEGF and MMP-9. Cells were seeded in single cultures (2x10^4^ cells/well in 200 μL for HT1080 or U937), with or without TNFα (1ng/mL), and increasing concentrations of recombinant human EMMPRIN or the IgG Fc fragment (Fc, at 200 ng/ml) were added as indicated. After 48h of incubation, supernatants were collected and concentrations of **(A, C)** MMP-9 and **(B, D)** VEGF were determined by ELISA (n=5-6 in all groups). The one-way ANOVA test, followed by the Dunn’s multiple *post-hoc* comparison test were used. The involvement of EMMPRIN was demonstrated by adding the blocking anti-EMMPRIN antibody (2 ng/ml) to a co-culture of HT1080 and U937 cells (2x10^4^ cell/well each) in the presence of TNFα (1 ng/mL). After 48h, the concentration of **(E)** VEGF and **(F)** MMP-9 were estimated by ELISA. The unpaired *t* test was used (n=7 in each group).

We next asked whether EMMPRIN is directly involved in the pro-angiogenic effects of the co-culture. To this end, we incubated each cell type alone with increasing amounts of human recombinant EMMPRIN protein. In both cell lines, the addition of TNFα was necessary to elevate MMP-9 levels, and a significant increase in MMP-9 level (by about 2 fold, p<0.05, **Figures 5A, C** **)** was observed upon adding a concentration of 500 ng/ml of recombinant EMMPRIN relative to the addition of TNFα alone to each cell line. On the other hand, TNFα had no influence on the expression of VEGF, and the addition of 500 ng/ml of recombinant EMMPRIN increased VEGF in HT1080 cells (about 2 fold, p<0.01), but not in U937 cells (**Figures 5B, D** **)**.

The neutralizing anti-EMMPRIN antibody was added to the two cell types co-cultured in the presence of TNFα, and after 48 hours of incubation, the accumulation of VEGF and MMP-9 in the supernatants was significantly reduced (by 1.8 and 1.4 respectively, p<0.05, **Figures 5E, F** **)**.

**Figure 6:**
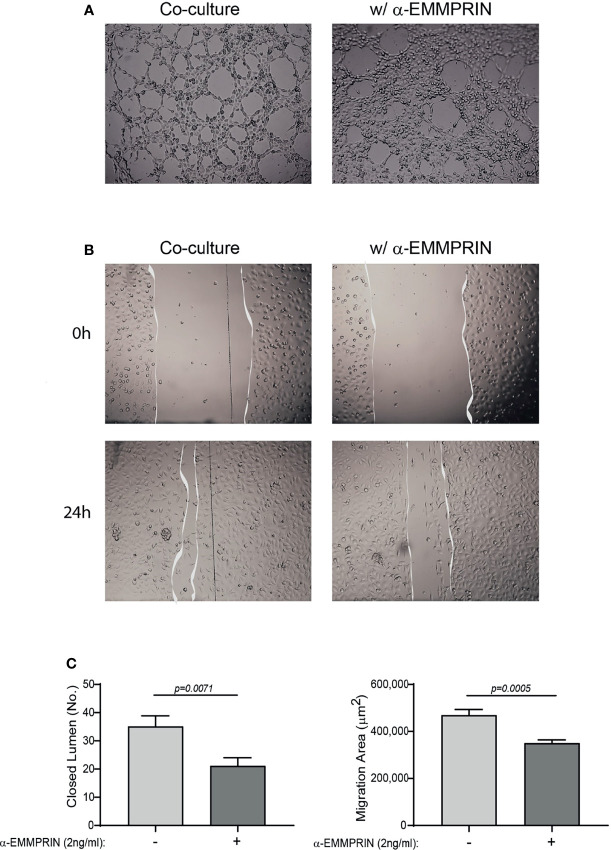
EMMPRIN mediates the angiogenic function of the co-culture. Supernatants derived from the HT1080 and U937 co-culture experiments were collected, and diluted 1:2 in full medium with or without the addition of blocking anti-EMMPRIN antibody (2 ng/mL). This mixture was added to EaHy926 endothelial cells (8x10^4^ cells/well in 200 μL) seeded on a Coulterx^®^ layer, and incubated for 6 hours. **(A)** Representative images (magnification ×4), and (**C**, left panel) quantification of the closed lumen tube-like structures. The unpaired t test was used (n=10). Alternatively, confluent monolayer of EaHy926 cells was scratched with a toothpick, detached cells were washed away, and cells were allowed to migrate for 24 hours to close the wound. **(B)** Representative images obtained at the beginning of the experiment (0 h) and after 24 hours (magnification ×4). (**C**, right panel) The area of the scratch at T24 was subtracted from the area at T0, to calculate the area endothelial cells migrated to. The unpaired *t* test was used (n=9).

Next, the overall contribution of EMMPRIN to the angiogenic potential of the supernatants was examined in functional *in vitro* assays. Conditioned media (CM) were collected from the TNFα-induced fibroblast-monocyte co-cultures, and EMMPRIN’s activity was neutralized by the addition of the anti-EMMPRIN antibody. These treated CM were then incubated with EaHy926 endothelial cells, and the effect was compared to the non-neutralized CM. Neutralization of EMMPRIN activity reduced the ability of endothelial cells to form tube-like structures (by 1.6 fold, p=0.071, **Figures 6A, C** **)**, or to migrate and close the gaps formed by a scratch (by 1.4, p=0.008, **Figures 6B, C** **)**.

### TCZ Regulates miR-146a-5p, Which in Turn Regulates EMMPRIN Expression in HT1080 Cells

**Figure 7:**
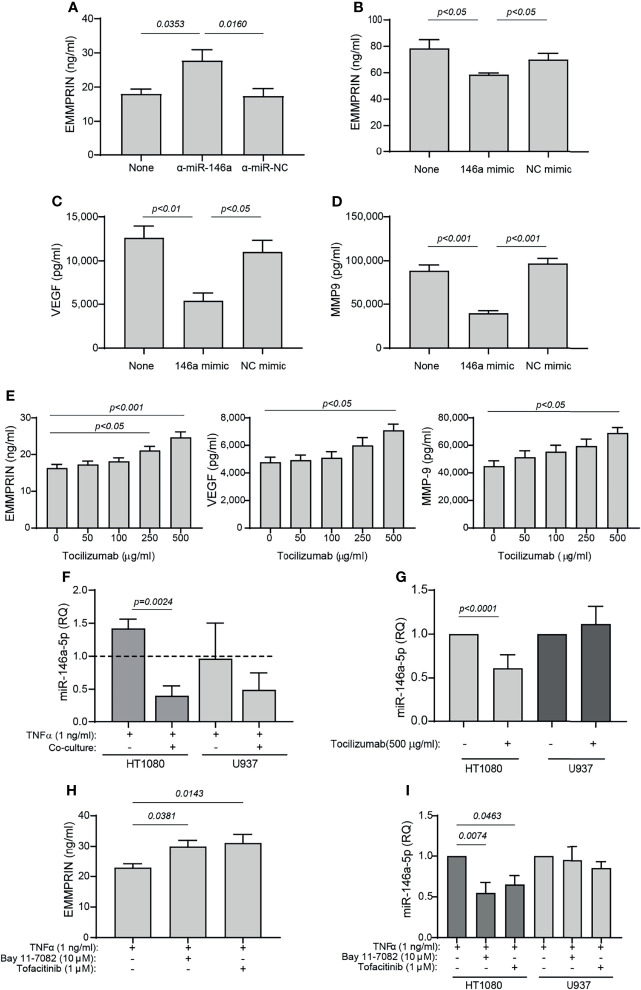
TCZ affects the expression levels of miR-146a and EMMPRIN. **(A–D)** HT1080 cells (10^4^ cells/well in 100 μL) were transfected with **(A)** anti-miR-146a-5p or its negative control, or with **(B–D)** miR-146a-5p mimic or its scrambled negative control (NC mimic). After transfection, cells were co-cultured with U937 cells with the addition of TNFα (1 ng/ml), and 48 hours later the concentrations of **(A, B)** EMMPRIN, **(C)** VEGF and **(D)** MMP-9 were determined by ELISA. The one-way ANOVA test, followed by the Bonferroni’s multiple *post-hoc* comparison test were used to assess the effects of the mimic and anti-miR-146a (n=5-6 for each group). **(E)** Increasing amounts of TCZ were added to HT1080 and U937 co-cultures with TNFα (1 ng/ml), and the concentrations of EMMPRIN, VEGF and MMP-9 were determined by ELISA. The one-way ANOVA test, followed by the *post-hoc* Dunn’s multiple comparison test were used (n=13 in each group). **(F)** Total RNA was extracted from HT1080 or U937 cells that were co-cultured with or without TNFα (1 ng/ml), and the expression levels of miR-146a-5p were determined in each of the cell types compared to single cultures without TNFα (dashed line). The unpaired student *t* test was used to compare between groups within each cell line. (n=5 in each group). **(G)** Expression levels of miR-146a-5p in co-cultures of HT1080 and U937 cells with or without the addition of TCZ (500 μg/ml) were determined in each cell type. The student *t* test was used to compare between the groups in each cell line (n=9-10 in each group). Lastly, HT1080 and U937 cells were incubated in co-culture for 48 hours in the presence of TNFα (1 ng/ml), with or without the NF-κB inhibitor Bay 11-7082 (10 μM) or the JAK/STAT inhibitor Tofacitinib (1 μM). **(H)** EMMPRIN concentrations and **(I)** miR-146a-5p expression levels were determined. The one-way ANOVA test, followed by the Bonferroni’s multiple *post-hoc* comparison test were used to assess the effects of the inhibitors on EMMPRIN and miR-146a expression (n=5).

Both miR-146a-5p and miR-150-5p were elevated in the serum of RA patients following 4 months of TCZ treatment. Since we have previously demonstrated that miR-146a-5p regulates EMMPRIN expression in tumor cells, we chose to focus on this miRNA and to explore whether it regulates EMMPRIN expression in fibroblasts and monocytes, and whether TCZ treatment affects EMMPRIN and angiogenesis through this regulatory pathway. We evaluated the expression of miR-146a-5p in each cell type in single cultures and in co-cultures in the presence of TNFα, compared to the single cultures without TNFα, which served as calibrators (indicated by the dashed line). For HT1080 cells, the co-culture reduced the levels of miR-146a-5p expression (by 3.5 fold, p=0.0024), while U937 showed no significant change (**Figure 7F**).

Next, we asked whether overexpression of miR-146a-5p would affect the expression levels of EMMPRIN, VEGF and MMP-9. HT1080 cells were transfected either with the miR-146a-5p mimic or with the anti-miR-146a-5p, as well as with their respective negative controls, and after 24 hours the transfected cells were incubated in co-culture with TNFα. Transfection of the anti-miR-146a-5p increased EMMPRIN levels (by 1.53 fold, p<0.05), whereas the miR-146-5p mimic reduced EMMPRIN, VEGF and MMP-9 levels (by 1.3, 2.3 and 2.2 fold, respectively, p<0.05, **Figures 7A–D**), and the negative controls did not differ from the co-culture with the non-transfected cells.

To assess the effects of TCZ on angiogenesis potential, we added increasing amounts of the drug to the co-cultured cells with TNFα, and observed that EMMPRIN, VEGF and MMP-9 were all increased (by 1.5 fold, p<0.05) at a concentration of 500 μg/ml relative to co-cultured cell without the drug (**Figure 7E**). Lastly, we show that TCZ at 500 μg/ml reduced miR-146a-5p expression levels in the HT1080 cells (by 1.6 fold, p<0.001), but not in the U937 cells.

To ask whether the NF-κB or the STAT pathways are involved in the regulation of EMMPRIN and miR-146a-5p expression, we co-cultured the HT1080 and U937 cells in the presence of TNFα and added either the NF-κB inhibitor Bay 11-7082 or the JAK/STAT inhibitor tofacitinib. We show that each of these inhibitors increased the expression of EMMPRIN in the supernatants of the co-cultured cells, relative to the control without the inhibitors (by about 1.3 fold, p<0.05), whereas the expression of miR-146a-5p was reduced by the inhibitors only in the HT1080 cells and not in the U937 cells (by 1.5-1.8 fold, p<0.05, **Figures 7H, I** **)**.

### TCZ Inhibits miR-146a-5p and Increases EMMPRIN Expression in Co-Cultured HT1080 and U937, Regardless of the Stimulation of the Cells With Either TNFα or IL-6

**Figure 8:**
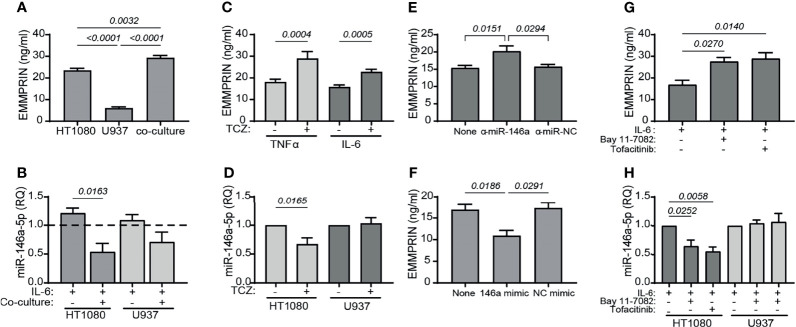
IL-6 affects EMMPRIN and miR-146-5p expression similarly to TNFα. **(A, B)** HT1080 and U937 cells (3x10^5^ cells in 800 μL) were co-cultured for 48h in the presence of recombinant IL-6 (20 ng/ml), and **(A)** EMMPRIN concentrations or **(B)** miR-146a-5p expression levels in each cell types were determined. The one-way ANOVA test, followed by the Bonferroni’s multiple *post-hoc* comparison test were used (n=4). **(C, D)** HT1080 and U937 cells were co-cultured with **(C)** TNFα (1 ng/ml) or IL-6 (20 ng/ml), or **(D)** with IL-6 (20 ng/ml) only, and with or without the addition of TCZ (500 μg/ml), and the effects on **(C)** EMMPRIN concentrations or **(D)** miR-146a-5p expression levels in each cell type were measured. The one-way ANOVA test, followed by the Bonferroni’s multiple *post-hoc* comparison test were used (n=4). **(E, F)** HT1080 cells (10^4^ cells/well in 100 μL) were transfected with **(E)** anti-miR-146a-5p or its negative control (α-miR-NC), or with **(F)** miR-146a-5p mimic or its negative control (NC mimic). After transfection, cells were co-cultured with U937 cells with the addition of IL-6 (20 ng/ml), and 48h later the concentrations of EMMPRIN were determined. The one-way ANOVA test, followed by the Bonferroni’s multiple *post-hoc* comparison test were used to assess the effects of the mimic and anti-miR-146a (n=4-5 for each group). Lastly, **(G, H)** HT1080 and U937 cells (3x10^5^ cells in 800 μL) were incubated in co-culture for 48h in the presence of IL-6 (20 ng/ml) and with or without the NF-κB inhibitor Bay 11-7082 (10 μM) or the JAK/STAT inhibitor Tofacitinib (1 μM). The concentrations of **(G)** EMMPRIN and **(H)** miR-146a-5p expression levels were measured. The one-way ANOVA test, followed by the Bonferroni’s multiple *post-hoc* comparison test were used to assess the effects of the inhibitors on EMMPRIN and miR-146a-5p expression (n=5).

Essentially, TCZ is designed to inhibit the IL-6 pathway by blocking IL-6R signaling. We, however, used TNFα as the primary stimulus in this study, as it a strong inducer of both MMP-9 and IL-6 (**Figure S3**). However, to confirm that IL-6 in the absence of TNFα could also affect miR-146a-5p and EMMPRIN expression, we stimulated the co-cultured HT1080 and U937 cells with IL-6 and observed that, similar to the presence of TNFα but to a lesser degree, EMMPRIN secretion was increased (by 1.25 fold, p<0.01, **Figure 8A**), whereas the expression of miR-146a-5p was reduced only in the HT1080 cells and not in the U937 cells, relative to non-stimulated cells (by 1.9 fold, p<0.05, **Figure 8B**). TCZ further increased EMMPRIN secretion (by 1.4 fold, p<0.001, **Figure 8C**) and reduced miR-146a-5p expression only in the HT1080 fibroblast cells (by 1.5 fold, p<0.05, **Figure 8D**). The effect on EMMPRIN was mediated by miR-146a-5p, as inhibiting it with its antagomir anti-miR-146a or overexpressing it by transfecting HT1080 cells with the miR-146a-5p mimic resulted in significant increased or decreased amounts of secreted EMMPRIN, respectively (**Figures 8E, F**). Lastly, by using the NF-κB pathway inhibitor Bay 11-7082 or the JAK/STAT pathway inhibitor tofacitinib in the presence of IL-6, we show that EMMPRIN is increased (by about 1.6 fold, p<0.05, **Figure 8F**) and miR-146a-5p is reduced only in the HT1080 cells (by about 1.5 fold, p<0.05, **Figure 8G**), in a similar way to what we demonstrated in the presence of TNFα.

### Co-Culture of the Primary Monocyte-Enriched PBMC With the HT1080 Fibroblasts Increase EMMPRIN, VEGF and MMP-9, and Reduce Tsp-1, and Addition of TCZ Reduce miR-146a-5p and Increase EMMPRIN

**Figure 9:**
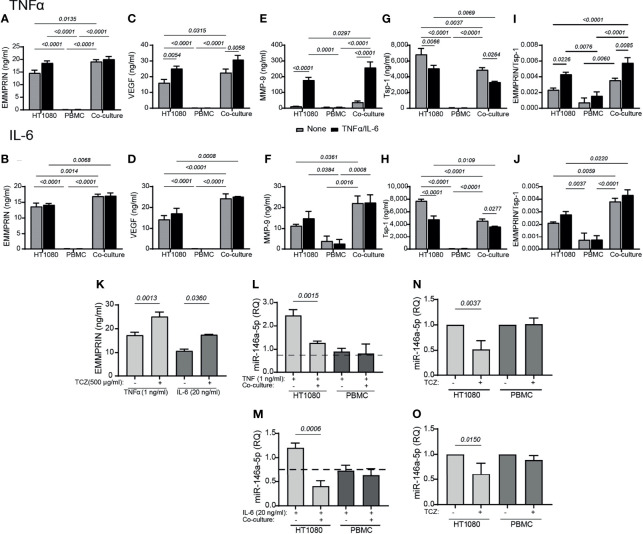
Primary monocyte-enriched PBMC co-cultured with HT1080 fibroblasts enhance the secretion of EMMPRIN, VEGF and MMP-9, and TCZ enhances EMMPRIN and reduces miR-146a-5p expression. HT1080 cells (8x10^4^ cells/well in 650 μL) were cultured alone or in co-culture with monocyte-enriched PBMCs at a ratio of 1:1, in the absence or presence of TNFα (1ng/mL, top panel) or IL-6 (20 ng/ml, lower panel). Supernatants were collected after 48h of incubation and the concentrations of **(A, B)** EMMPRIN, **(C, D)** VEGF, **(E, F)** MMP-9, and **(G, H)** Tsp-1 were determined by ELISA. **(I, J)** The EMMPRIN/Tsp-1 ratio was calculated for each repetition. The two-way analysis of variance (ANOVA) test was used to compare multiple groups, followed by the *post-hoc* Bonferroni’s multiple comparison test (n=4-5 in all groups). Co-cultured HT1080 cells and monocyte-enriched PMBC were incubated in the presence or absence of TCZ (500 μg/ml), and **(K)** their levels of secreted of EMMPRIN were determined. The unpaired student *t* test was used to compare between groups within each cell line (n=4-5 in each group). Total RNA was extracted from HT1080 or monocyte-enriched PBMC that were co-cultured with or without **(L)** TNFα (1 ng/ml) or **(M)** IL-6 (20 ng/ml) for 48 hours, and the expression levels of miR-146a-5p were determined in each of the cell types compared to single cultures without the cytokines (dashed line). The unpaired student *t* test was used to compare between groups within each cell line. (n=4-5 in each group). Lastly, the effects of TCZ on the expression levels of miR-146a-5p in each cell type in the presence of **(N)** TNF (1 ng/ml) or **(O)** IL-6 (20 ng/ml) were determined. The unpaired student *t* test was used to compare between groups within each cell line. (n=4-5 in each group).

As we have used two cell lines in our study that do not necessarily represent physiological conditions, we next wanted to confirm our main results with primary monocytes. We have isolated PBMC from healthy donors, and co-cultured them with the HT1080 cell line. Similar to the previous results of the co-cultured HT1080 and U937 cells, we show here that the levels of secreted EMMPRIN, VEGF and MMP-9 in HT1080 cells co-cultured with primary monocytes-enriched PBMC were increased after 48 hours of incubation relative to single HT1080 culture (**Figures 9A-F**). While IL-6 did not have an additional effect on the co-cultured cells, TNFα enhanced the secretion of MMP-9 and VEGF relative to unstimulated cells. In contrast, relative to the HT1080 single culture, the secreted levels of Tsp1 were decreased either by the co-culture or by the addition of the cytokines (**Figures 9G, H** **)**. Hence, the ratio EMMPRIN/Tsp-1 was increased by the co-culture relative to the single culture of HT1080 cells, and the addition of TNFα, but not IL-6, further increased it (**Figure 9I**). The co-culture in the presence of TNFα or IL-6 also reduced the expression of miR-146a-5p in the HT1080 cells, but not in the monocyte-enriched PBMC, relative to the HT1080 single culture (**Figures 9L, M**).

Similar to the previous results, the addition of TCZ enhanced EMMPRIN secretion and reduced miR-146a-5p expression in the HT1080, but not in the monocyte-enriched PBMC (**Figures 9K, N, O**). Thus, the monocyte-enriched PBMC confirmed that primary monocytes behave in a similar way to the U937 monocytic-like cell line, and promote pro-angiogenic changes in the HT1080 fibroblast cell line.

## Discussion

Angiogenesis is an important process in the pathophysiology of RA (34), but the mechanisms regulating it are yet unclear. TCZ is a biologic agent indicated for the treatment of RA (2), but its effects on pathological angiogenesis have not been sufficiently studied. Here we demonstrate that EMMPRIN (known to be a pro-angiogenic factor in the tumor microenvironment (35)) is involved in angiogenesis in RA patients and in a co-culture of fibroblasts and monocytes *in vitro*. Furthermore, we demonstrate that TCZ affects the angiogenic process, at least partially, through its effects on pro-angiogenic factors, particularly EMMPRIN and its regulator miR-146a-5p. We also demonstrate that the ratio between EMMPRIN and Tsp-1 levels is a useful measure of the angiogenic state in RA patients.

The effects of TCZ on our RA patient cohort concurred with the known effects of TCZ, showing clinical improvement in arthritis, and causing reductions in DAS28 scores and high sensitivity CRP levels. Also, in accordance with previous observations, IL-6 serum levels increased in RA patients following initiation of TCZ treatment (**Figure S2**) (2, 36).

We show here that after 4 months of treatment, TCZ reduced EMMPRIN serum levels as well as the EMMPRIN/Tsp-1 ratio (**Figure 1**). With the help of the functional tube-formation assay, we were able to demonstrate a direct effect of EMMPRIN on endothelial cells (**Figure 6**). However, despite the reduction in EMMPRIN, with its known ability to induce VEGF and MMPs (15, 35), no parallel reduction in the serum levels of VEGF or MMP-9 occurred in the sera of RA patients (**Figure 1**). This finding may be explained by the presence of alternative signaling pathways to that of EMMPRIN which may induce VEGF and MMP-9 secretion, such as TNFα, a known inducer of MMP-9 (33) or tissue hypoxia, a known inducer of VEGF (37, 38). Thus, the inhibition of the IL-6 signaling pathway by TCZ may not be sufficient to reduce the serum concentrations of these mediators in the synovial microenvironment, which is rich in pro-inflammatory cytokines.

When setting up the *in vitro* system, we chose to stimulate the cells with TNFα, a known inducer of both MMP-9 and IL-6. We show that TNFα induced IL-6 levels in the HT1080 cells, and reduced the shedding of IL-6R expression in U937 cells (**Figure S3**). Thus, the ability of TCZ to inhibit IL-6 signaling could be mimicked in our *in vitro* system. Since TCZ is an inhibitor of IL-6 signaling and TNFα might have different effects, we further verified these results by repeating the experiment in the presence of IL-6 alone (**Figure 8**), and demonstrated a similar ability of TCZ to inhibit miR-146a-5p expression levels and enhance EMMPRIN secretion. However, it is still unclear whether TNFα acts directly on miR-146a-5p and EMMPRIN, or whether it works through its ability to induce IL-6. The findings in our study should be further verified by using primary human synoviocytes, however, collection of synovial fluid was not part of our approved protocol, and we could not isolate such cells.

Next, we demonstrate that EMMPRIN is directly involved in the regulation of angiogenesis using an *in vitro* co-culture system, as its levels were increased in the media of co-cultured fibroblasts and monocytes together with those of VEGF and MMP-9 (**Figure 4**), and the anti-EMMPRIN antibody reduced these levels while recombinant EMMPRIN increased them (**Figure 5**). This involvement was further established by its direct effects on migration, proliferation, and tube-formation of the endothelial cells in the scratch and tube formation assays, and the ability of an anti-EMMPRIN antibody to reduce them (**Figure 6**). Of note, the obvious inconsistency between the unchanged serum levels of VEGF and MMP-9 in the RA patients (**Figure 1**) and their elevated levels in the *in vitro* system may suggest the involvement of additional factors in their regulation, including other cell types and multiple cytokines, which may not have been present in the isolated *in vitro* system. This premise is also supported by the fact that monocyte-enriched PBMC produced similar results to those of the U937 cells when co-cultured with the HT1080 cells (**Figure 9**). Such differences between the *in vivo* and *in vitro* systems are exemplified by reduction in the pro-angiogenic factor TGFβ in the patients after TCZ treatment, whereas in the *in vitro* setting TGFβ levels were unchanged by TCZ treatment (**Figure S4**).

Although no difference was detected in EMMPRIN, VEGF, MMP-9 or Tsp-1 serum levels between patients who responded well to TCZ treatment and those who were unresponsive to therapy, the ratio between EMMPRIN and Tsp-1 was reduced in the responding patients compared to the non-responders (**Figure 2**). This suggest that use of the EMMPRIN/Tsp-1 ratio, which takes into account small changes in the balance between pro- and anti-angiogenic factors, might be a reliable way to assess the angiogenic status in patients. However, additional studies are necessary to establish the validity of this proposed parameter in assessing the effects of conventional or biologic DMARDs on angiogenesis in RA patients.

Among the pro-angiogenic factors we tested, NGAL was significantly elevated in TCZ-treated patients, as well as in responding patients relative to non-responders (**Figures 1**, **2**). NGAL is known to form heterodimers with MMP-9, thus protecting the latter from degradation. In addition, NGAL has been shown to regulate VEGF expression and to promote angiogenesis (39). Previous reports demonstrated higher levels of NGAL in the serum of RA patients compared to healthy controls, suggesting that it promotes angiogenesis (40). In this regard, the observation of elevated levels of NGAL in RA patients after TCZ administration is contrary to the general anti-angiogenic effects mediated by TCZ. However, notably, NGAL plays many other pleiotropic roles unrelated to its role in angiogenesis, such as in protecting against bacterial infection by acting as an iron-carrying protein, in modulating oxidative stress, in promoting neutrophil chemotaxis, and in regulating thermogenesis and lipid metabolism by acting as an adipokine (10, 30). Moreover, although NGAL normally protects MMP-9 from degradation, we did not observe any elevation in MMP-9 serum levels in RA patients. This finding further supports the notion that the primary role played by NGAL in RA is unrelated to angiogenesis, and that its elevation after initiation of TCZ treatment may be related to its role as an adipokine. This possibility must be carefully explored in a follow-up study.

From the nine miRNAs that we examined, we observed that only miR-146a-5p and miR-150-5p were elevated after 4 months of treatment (**Figure 3**). Previous studies have implicated these two miRNAs in the inflammation that is driving RA. The long non-coding RNA LINC01197 which exhibits low levels in RA patients, normally acts as a sponge that binds miR-150-5p, thereby leading to enhanced thrombospondin-2 levels and reduced inflammation (41). In agreement with our findings, previous studies have shown increased levels of miR-146a-5p in peripheral blood mononuclear cells (PBMC) derived from RA patients (42), and the increased miR-146a-5p levels found in synovial fluid and in PBMC derived from RA patients were linked to decreased apoptosis in CD4^+^ T cells derived from RA patients (43). Outside the context of RA, TCZ was shown to increase the serum levels of miR-146a-5p in COVID-19 patients, and its levels could predict response to the drug (44). However, we are unaware of any other previously published studies examining the effects of TCZ on the expression of the particular miRNAs we chose to examine in our study, and only one study demonstrated an increase in the level of a different miRNA - miR-148a - by TCZ in neutrophils isolated from RA patients *in vitro* (45).

Since we have already previously shown that miR-146a-5p participates in the regulation of EMMPRIN expression in tumor cells (26, 27), we suspected that this miRNA was also involved in EMMPRIN regulation in fibroblasts, and therefore focused on this miRNA in our *in vitro* experiments. We demonstrate that transfection of the fibroblast cell line HT1080 with the miR-146a-5p mimic resulting in overexpression of miR-146a-5p in the HT1080 cells, reduced the secretion of EMMPRIN, and subsequently of VEGF and MMP-9, implicating this miRNA in the regulation of EMMPRIN in fibroblasts (**Figures 7B–D**). In contrast, inhibiting miR-146a-5p activity by transfecting the HT1080 cells with its antagomir increased EMMPRIN secretion (**Figure 7A**), whether the cells were stimulated with TNFα or with IL-6. Thus, miR-146a-5p is involved in the regulation of EMMPRIN expression in the fibroblast cell line, directly or indirectly by affecting other regulators of EMMPRIN.

We note that TCZ decreased miR-146a-5p levels and increased EMMPRIN levels in fibroblasts *in vitro* (**Figures 7E, G**, **8C, D**, **9N, O**), whereas it increased miR-146a-5p levels and decreased EMMPRIN levels in the serum samples from treated RA patients (**Figures 1**, **3**). These inconsistencies may be explained by the difference between the *in vitro* and *in vivo* systems or by differing effects exerted by TCZ on different cell types. Indeed we could observe that the effects of TCZ on miR-146a-5p were specific to the HT1080 fibroblasts, and TCZ had no significant effect on the miR-146a-5p levels in the monocytic U937 cells or in the monocyte-enriched PBMCs (**Figures 7G**, **8D**, **9N, O**). Alternatively, the serum may reflect the state in the synovium, where interactions between fibroblasts and many other cell types may generate a balance different from that observed in the *in vitro* co-culture system involving only two cell-lines. However, despite these inconsistencies, both the *in vivo* and the *in vitro* systems reflect a strong link between miR-146a-5p, EMMPRIN and the angiogenic process, and demonstrate the ability of TCZ to intervene in this process.

The induction of miR-146a-5p is mostly attributed to stimulators activating the NF-κB pathway, such as TNFα and IL-6 (46, 47), and accordingly its levels have been shown to increase in RA patients (47). Therefore, the presence of TNFα or IL-6 in our *in vitro* system can explain the upregulation of miR-146a-5p compared to non-stimulated cells (**Figure 7F**), and the inhibitory effect of TCZ on the expression of miR-146a-5p (**Figures 7G**, **8D**, **9N, O**) might suggest a disruption of this pathway. This assumption is supported by the cooperation between TNFα-induced NF-κB and the JAK/STAT3 pathway which has recently been demonstrated in brain pericytes (48), and by the inhibitory effects of TCZ on NF-κB in a rat model of sepsis (49). Furthermore, by inducing IL-6, TNFα could indirectly activate the JAK/STAT pathway. To explore whether the NF-κB or the JAK/STAT pathways are involved in the regulation on miR-146a-5p expression in our system, we used the NF-κB inhibitor Bay 11-7082 and the JAK/STAT inhibitor tofacitinib, and observed an increase in EMMPRIN secretion and a decrease in miR-146a-5p expression, regardless of whether TNFα or IL-6 were used to stimulate the cells (**Figures 7H, I**, **8G, H**). Thus, we suggest that TCZ helps regulate the TNFα/IL-6-induced expression of miR-146a-5p through interference with both the NF-κB and JAK/STAT pathways, and consequently controls the expression of EMMPRIN and thereby of angiogenesis. Further investigation is required to map the exact nature of this interference.

## Conclusion

In summary, we establish an important role for EMMPRIN in mediating pro-angiogenic signals in RA patients and demonstrate a strong link between miR-146a-5p expression and the regulation of EMMPRIN secretion. Importantly, we show that TCZ reduces the angiogenic potential in RA patients, and we suggest that this is partially due to the ability of TCZ to interfere with the expression of miR-146a-5p, leading to changes in EMMPRIN levels. We also suggest that the ratio between EMMPRIN and Tsp-1 may reflect the angiogenic status in RA patients more accurately than any one factor alone.

## Data Availability Statement

The original contributions presented in the study are included in the article/**Supplementary Material**. Further inquiries can be directed to the corresponding authors.

## Ethics Statement

The studies involving human participants were reviewed and approved by Institutional Review Board at Carmel Medical Center (CMC-0018-11). The patients/participants provided their written informed consent to participate in this study.

## Author Contributions

MS, ES, AK, and LZ performed the experiments. JF, TG, AH, ME, IR, LK, and DZ recruited the patients. JF secured the funding. TG, JF, AH, ME, IR, and LK reviewed and edited the paper. MR and DZ designed the work, analyzed and interpreted the data, and drafted the paper. All authors contributed to the article and approved the submitted version.

## Funding

This research was partially supported by a young physician scholarship from the Israeli Rheumatology Association.

## Conflict of Interest

The authors declare that the research was conducted in the absence of any commercial or financial relationships that could be construed as a potential conflict of interest.

## Publisher’s Note

All claims expressed in this article are solely those of the authors and do not necessarily represent those of their affiliated organizations, or those of the publisher, the editors and the reviewers. Any product that may be evaluated in this article, or claim that may be made by its manufacturer, is not guaranteed or endorsed by the publisher.

## Supplementary Material

Supplementary Figure S1 TCZ treatment does not change U6 expression in RA patients. The expression levels U6 and miR-16 manifested by the C_T_ values of patients before and 4 months following initiation of TCZ treatment were determined by qPCR as described (n=37). The change in the expression of U6 was lower relative to the change in miR-16, and therefore, it was chosen for the normalization of the rest of the studied miRNAs.

Supplementary Figure S2 TCZ increases serum levels of IL-6 and decreases those of hsCRP. Box plot representing the concentrations of **(A)** IL-6 and **(B)** hsCRP in the serum of RA patients before and after 4 months of TCZ treatment in comparison to healthy volunteers (controls). Patients were stratified into “responders” and “non-responders” according to the change in their DAS-28 score (≧1.2 vs <1.2, respectively), and the effects of TCZ on the concentrations of **(C)** IL-6 and **(D)** hsCRP are indicated. The non-parametric Mann-Whitney test was used to compare between the concentrations of each of the cytokines before and 4 months after TCZ treatment (n=40).

Supplementary Figure S3 TNFα induces IL-6 in HT1080 cells and decreases IL-6R in U937 cells. HT1080 cells (4x10^5^ cells) were cultured alone or in co-culture with U937 monocytes at a ratio of 1:1, in the absence or presence of TNFα (1ng/mL) or TCZ (500 μg/ml). Supernatants were collected after 48h of incubation and the concentrations of **(A)** IL-6, and **(B)** IL-6R were determined by ELISA. The one-way ANOVA test followed by the Bonferroni’s multiple *post-hoc* comparison test was used (n=6 in all groups).

Supplementary Figure S4 TGFβ is reduced after TCZ treatment in RA patients, but not in the *in vitro* co-culture. **(A)** HT1080 cells (4x10^5^ cells/well in 800 μL) were cultured alone or in co-culture with U937 monocytes at a ratio of 1:1, in the absence or presence of TNFα (1ng/mL). Supernatants were collected after 48h of incubation and the concentrations of TGFβ were unchanged. **(B)** Box plot representing the concentrations of angiogenic mediators in the serum of RA patients before and 4 months after initiation of TCZ treatment, as determined in duplicates for each sample.

## Abbreviations

bDMARDs, biologic disease modifying anti-rheumatic drugs; cDMARDs, conventional disease modifying anti-rheumatic drugs; CRP, C-reactive protein; EMMPRIN/CD147, extracellular matrix metalloproteinase inducer; FLS, fibroblast-like synoviocytes; IL, interleukin; JAK, Janus Kinase; MAPK, mitogen activated protein kinase; miRNA, microRNA; MMPs, matrix metalloproteinases; MVD, mean vessel density; NF-κB, nuclear factor kappa-light-chain-enhancer of activated B cells; NGAL, neutrophil gelatinase associated lipocalin (lipocalin-2); RA, Rheumatoid arthritis; SAA, serum amyloid A; STAT, signal transducers and activators of transcription; VEGF, vascular endothelial growth factor; TCZ, tocilizumab; TNFα, tumor necrosis factor alpha; Tsp-1, thrombospondin-1.
