# Supplementary figures and images for: Tocilizumab (TCZ) Decreases Angiogenesis in Rheumatoid Arthritis Through Its Regulatory Effect on miR-146a-5p and EMMPRIN/CD147
Source: Front Immunol. 2021 Dec 15;12:739592. doi: 10.3389/fimmu.2021.739592 (PMC8714881; doi:10.3389/fimmu.2021.739592)

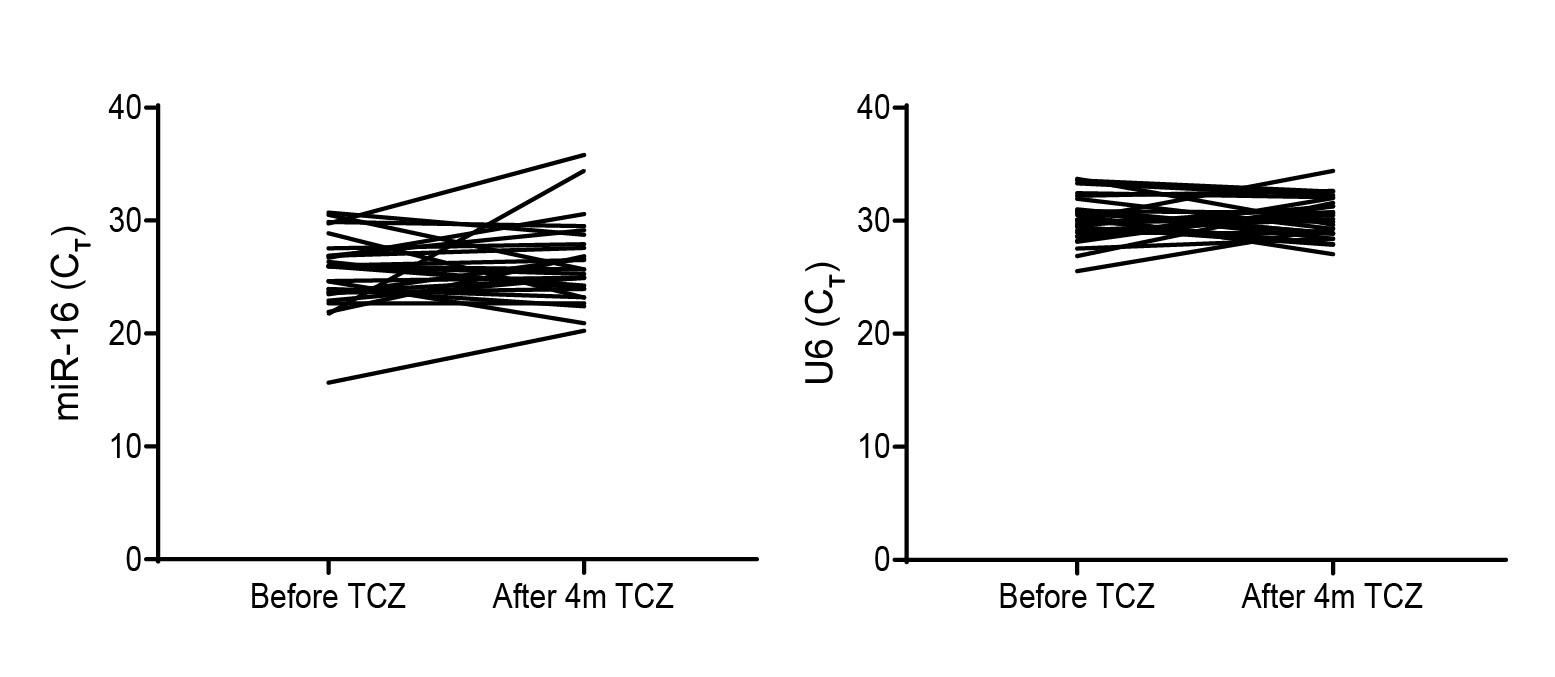

Supplement: Supplementary Figure S1 — TCZ treatment does not change U6 expression in RA patients. The expression levels U6 and miR-16 manifested by the CT values of patients before and 4 months following initiation of TCZ treatment were determined by qPCR as described (n=37). The change in the expression of U6 was lower relative to the change in miR-16, and therefore, it was chosen for the normalization of the rest of the studied miRNAs. [file Image_1.jpeg]

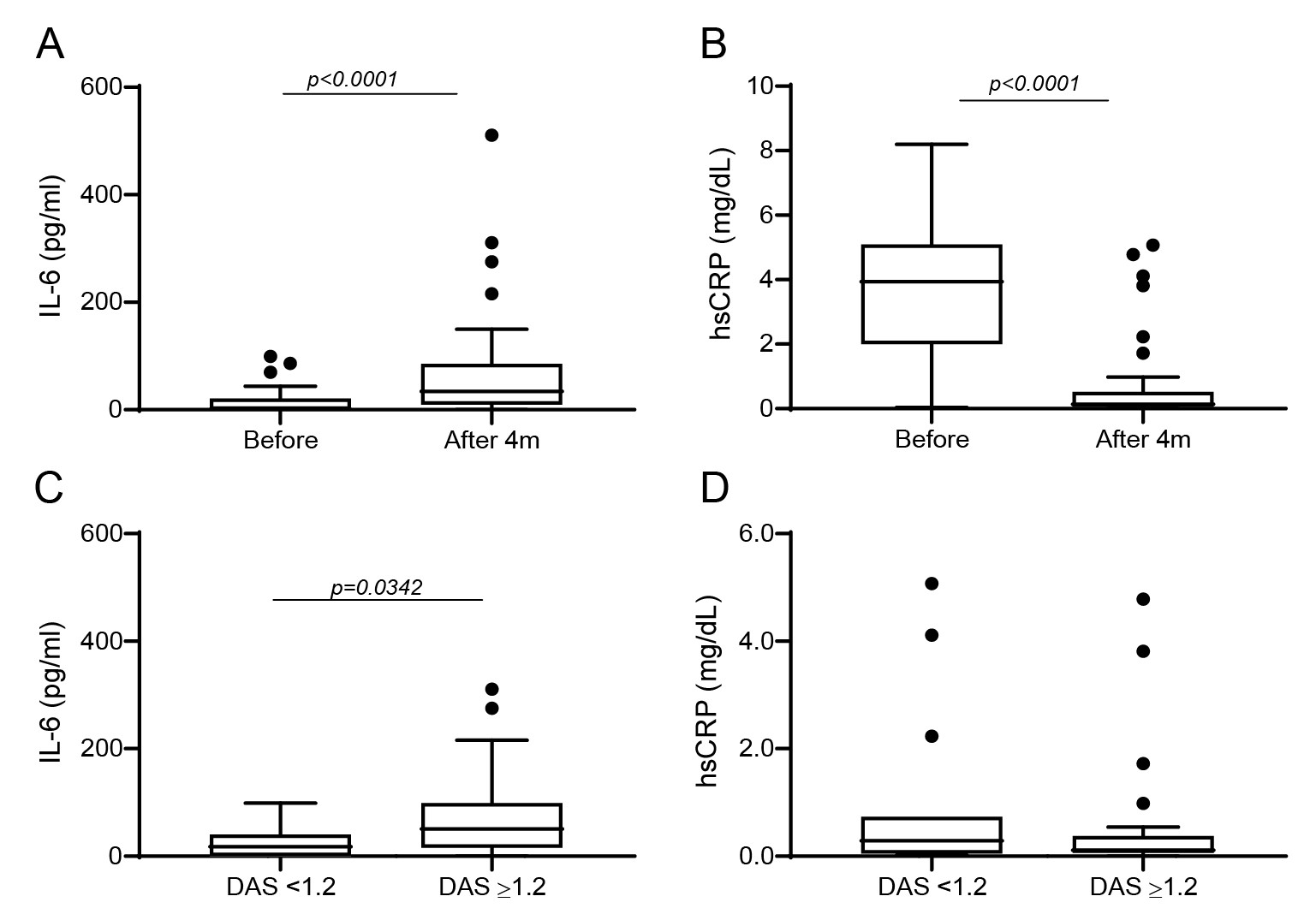

Supplement: Supplementary Figure S2 — TCZ increases serum levels of IL-6 and decreases those of hsCRP. Box plot representing the concentrations of (A) IL-6 and (B) hsCRP in the serum of RA patients before and after 4 months of TCZ treatment in comparison to healthy volunteers (controls). Patients were stratified into “responders” and “non-responders” according to the change in their DAS-28 score (≧1.2 vs <1.2, respectively), and the effects of TCZ on the concentrations of (C) IL-6 and (D) hsCRP are indicated. The non-parametric Mann-Whitney test was used to compare between the concentrations of each of the cytokines before and 4 months after TCZ treatment (n=40). [file Image_2.jpeg]

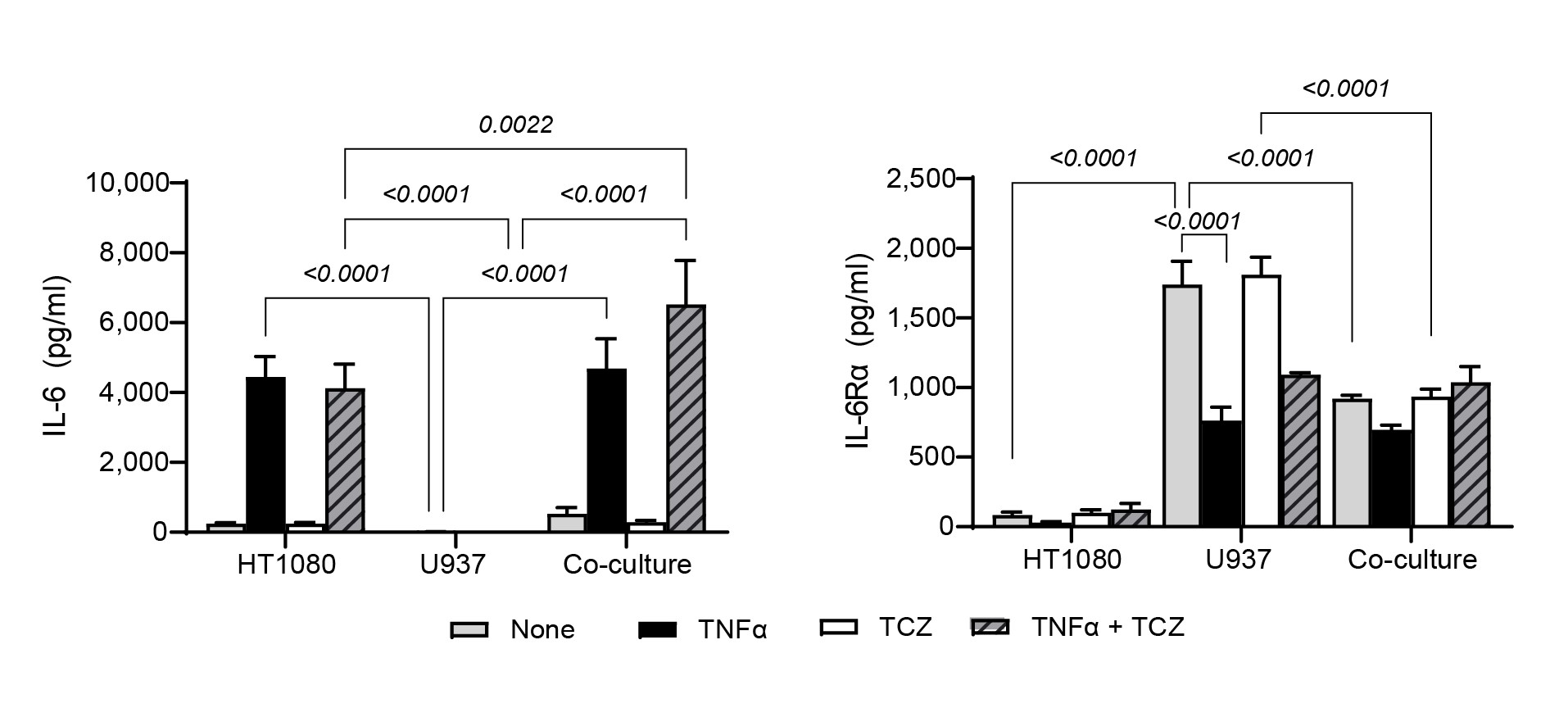

Supplement: Supplementary Figure S3 — TNFα induces IL-6 in HT1080 cells and decreases IL-6R in U937 cells. HT1080 cells (4x10^5^ cells) were cultured alone or in co-culture with U937 monocytes at a ratio of 1:1, in the absence or presence of TNFα (1ng/mL) or TCZ (500 μg/ml). Supernatants were collected after 48h of incubation and the concentrations of (A) IL-6, and (B) IL-6R were determined by ELISA. The one-way ANOVA test followed by the Bonferroni’s multiple post-hoc comparison test was used (n=6 in all groups). [file Image_3.jpeg]

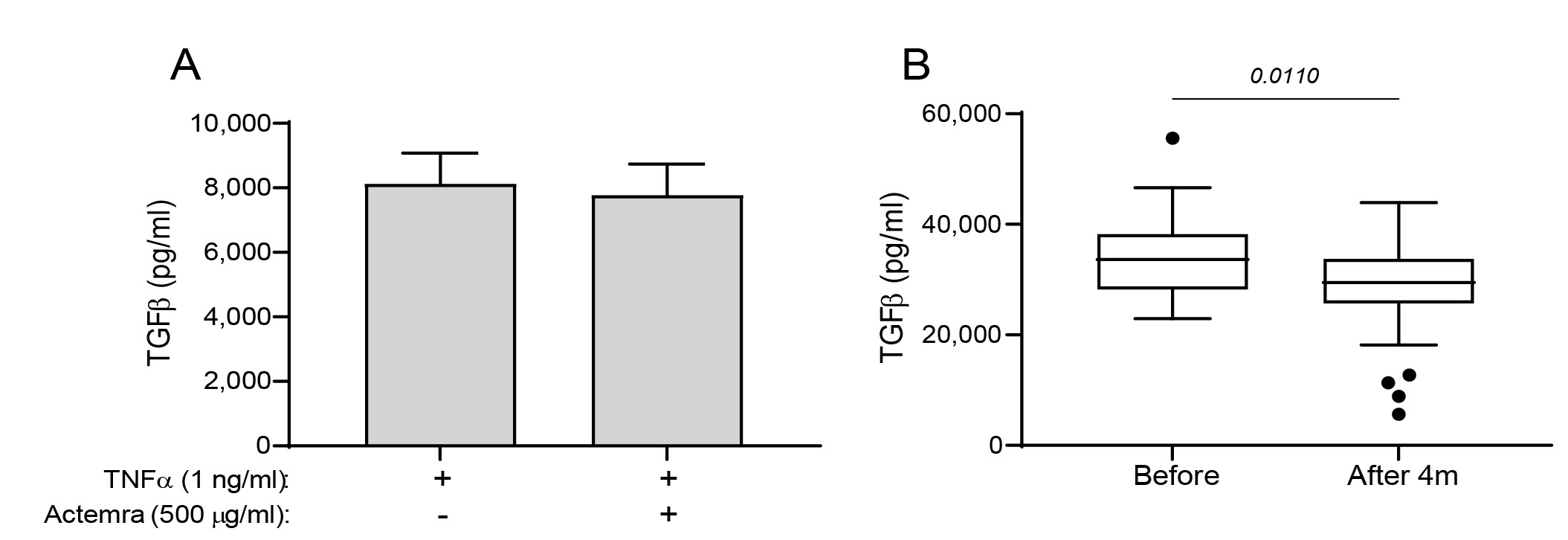

Supplement: Supplementary Figure S4 — TGFβ is reduced after TCZ treatment in RA patients, but not in the in vitro co-culture. (A) HT1080 cells (4x10^5^ cells/well in 800 μL) were cultured alone or in co-culture with U937 monocytes at a ratio of 1:1, in the absence or presence of TNFα (1ng/mL). Supernatants were collected after 48h of incubation and the concentrations of TGFβ were unchanged. (B) Box plot representing the concentrations of angiogenic mediators in the serum of RA patients before and 4 months after initiation of TCZ treatment, as determined in duplicates for each sample. [file Image_4.jpeg]
